# Editorial: Advances in Percutaneous Coronary Intervention

**DOI:** 10.3389/fcvm.2022.914487

**Published:** 2022-06-27

**Authors:** Angelo Silverio, Giuseppe De Luca, Giovanna Sarno, Gennaro Galasso

**Affiliations:** ^1^Department of Medicine, Surgery and Dentistry, University of Salerno, Salerno, Italy; ^2^Clinical and Experimental Cardiology, AOU Sassari, Sassari, Italy; ^3^Department of Medical Sciences, Cardiology and Uppsala Clinical Research Center, Uppsala University, Uppsala, Sweden

**Keywords:** percutaneous coronary intervention, outcome, acute coronary syndrome, coronary artery disease, myocardial infarction

There have been significant advances in percutaneous coronary intervention (PCI) with new devices and drugs, new technology applications, and new indications for revascularization based on the latest evidence from large randomized or observational studies. This progress has contributed not only to a higher rate of successful procedures, but also to sustained long-term results and improved patient outcomes.

Many authors contributed to this special issue. Their articles cover different areas of interest for interventional cardiologists and offer insights into the implementation of diagnosis and treatment of patients with coronary artery disease (CAD).

In the last decade, the invasive functional assessment of coronary lesions to identify critical coronary artery stenosis (CAST) has become available in most laboratories and can be used to integrate the luminographic information of coronary angiography. Fractional flow reserve (FFR) or related indices may reduce the number of unnecessary PCI, with a reduction of costs and potential benefit in terms of quality of life and outcome compared to the angiography-guided standard approach (1–3). However, this may be complicated and not always feasible in acute coronary syndromes due to the presence of multivessel disease, patient status, and setting reasons. In their review article, Haley et al. discuss the challenges of invasive functional assessment in patients with acute coronary syndrome, and encompass the principles, tips and tricks, advantages, and limitations of virtual FFR (vFFR) in this patient population. The vFFR does not need a pressure wire and uses only coronary angiography images processed by using computational fluid dynamics. The authors report in detail the systems currently available to calculate vFFR, all requiring manual image correction prior to computational fluid dynamics processing, and emphasize that the validity of measurements depends mainly on good angiographic images (almost half of angiograms are unsuitable for processing). Beyond these limitations, vFFR offer many advantages compared to FFR or related methods, and appears very attractive in patients with ACS as it provides combined functional and anatomical assessment of intermediate lesions in a single time, without pressure wire or pharmacologic hyperemia. vFFR is still poorly available in most catheterization laboratories and the evidence supporting its use is largely based on retrospective studies.

A complemental issue covered by this collection was the non-invasive assessment of CAST for reducing the proportion of patients who undergo coronary angiography, but then have no evidence of CAST. Regional LV function is highly dependent on subendocardial blood flow and CAST may impair regional function within the ischaemic risk area. The non-invasive echocardiographic assessment of the resulting systolic impairment may be challenging since parameters of longitudinal deformation are highly dependent on the loading conditions and this could limit their discriminative accuracy for CAST (4–7). Sabatino et al. in their study including 80 consecutive patients with CAD investigated the ability of a novel non-invasive echocardiographic method to estimate myocardial work (MW) as an index to predict the presence of CAST. They found that regional MW efficiency, which is the ratio between constructive work and the sum of constructive and wasted work, was significantly reduced within the myocardial segments underlying CAST compared to non-target segments, and showed high discriminative accuracy for non-invasive detection of CAST (AUC = 0.92). Noteworthy, this result was not observed for the other tested parameters including longitudinal strain assessed by speckle-tracking echocardiography and post-systolic shortening index, suggesting that regional MW efficiency outperforms other echocardiographic parameters for this application.

This study suggests that MW efficiency has the potential to identify myocardial segments underlying CAST, and could represent a valid implementation of the sole strain measurements. Noteworthy, this result was limited by the small sample size and cannot be generalized to patients with different pre-test probability for CAST, which emphasizes the importance of future confirmatory studies.

Continuing with our special issue, Liang and Gu performed a pooled analysis of 628 patients prospectively enrolled in the serial Disrupt CAD trials I-IV, which evaluated the performance of intravascular lithotripsy (IVL) for treatment of severely calcified coronary lesions.

The authors reported a very low incidence of major adverse cardiac events, a high rate of procedural success (93%), and described circumferential and transmural calcium fracture assessed by optical coherence tomography as the underlying mechanism of action IVL. Also, the rate of procedural success, angiographic success, and stent delivery reported in the Disrupt CAD trials were similar to that reported by the ROTAXUS and PREPARE-CALC, which evaluated rotational atherectomy (8, 9). The authors pointed out the utility of IVL for the preparation of heavy calcified coronary lesions, the ease of use compared with atherectomy-based techniques, end the short learning curve. However, there are still no data supporting the advantage of IVL over the other methods, and head-to-head studies are warranted to guide treatment decisions toward a patient-tailored strategy.

This special issue concludes with an article by Li et al., who reported a real-world multicentre experience on 3,049 patients undergoing PCI with bivalirudin anticoagulation treatment. Although the use of bivalirudin during PCI of patients with acute coronary syndromes has been downgraded by current guidelines (10), the authors reported a substantially low rate of bivalirudin-related adverse drug reactions (ADRs) and no deaths, suggesting good tolerance and safety of this anticoagulant. The authors identified age, renal function, and the CRUSADE score as independent predictors of ADRs and propose their implementation for prognostic stratification in this patient population.

This article collection covers many aspects of interventional cardiology without forgetting what is our primary goal: update our knowledge to better serve our patients. The safety and efficacy of PCI continue to evolve with the development of newer devices, techniques, and pharmacological agents. This innovation starts with the ideas of individuals and real-world experiences, and gradually moves up the levels of the evidence pyramid.

## Author Contributions

All authors listed have made a substantial, direct, and intellectual contribution to the work and approved it for publication.

## Conflict of Interest

The authors declare that the research was conducted in the absence of any commercial or financial relationships that could be construed as a potential conflict of interest.

## Publisher's Note

All claims expressed in this article are solely those of the authors and do not necessarily represent those of their affiliated organizations, or those of the publisher, the editors and the reviewers. Any product that may be evaluated in this article, or claim that may be made by its manufacturer, is not guaranteed or endorsed by the publisher.
